# Deep Learning for Diagnosis of Choroideremia and *USH2A*-Associated Rod–Cone Dystrophy Using Macular OCT Volumes

**DOI:** 10.1016/j.xops.2026.101378

**Published:** 2026-08-20

**Authors:** Kevin Mairot, Isabelle Meunier, Béatrice Bocquet, Natacha Stolowy, Prithvi Ramtohul, Xavier Zanlonghi, Danièle Denis, Thierry David, Frederic Matonti, Laurent Udo Perrinet

**Affiliations:** 1 Institut de Neurosciences de la Timone, Aix Marseille Univ, CNRS, Marseille, France; 2 Aix Marseille University, CNRS, Marseille, France; 3 Department of Ophthalmology, University North Hospital of Marseille, Sensgene Care Network, Marseille, France; 4 National Reference Centre for Inherited Sensory Diseases, University of Montpellier, Montpellier University Hospital, Sensgene Care Network, ERN-EYE Network, Montpellier, France; 5 Institute for Neurosciences of Montpellier (INM), University of Montpellier, INSERM, Montpellier, France; 6 Department of Ophthalmology, Rennes University Hospital, Rennes, France; 7 Retinal Surgery Department, Centre Monticelli Paradis, Marseille, France

**Keywords:** Choroideremia, Deep learning, Inherited retinal dystrophy, USH2A

## Abstract

**Objective:**

To develop and validate deep learning models to differentiate choroideremia (CHM), *USH2A*-associated rod–cone dystrophy, and healthy controls using macular OCT volumes.

**Design:**

Retrospective diagnostic study with external validation.

**Subjects:**

Two hundred sixty-two participants contributed 535 macular OCT volumes acquired on a Heidelberg Spectralis (10 165 B-scans): 99 participants had *CHM*, 96 had *USH2A*-associated disease, and 67 were healthy controls.

**Methods:**

Volumes acquired between 2012 and 2021 were standardized to 19 B-scans and split at the patient level into training (76%), validation (12%), and test (12%) sets. Three strategies were compared: (1) a slice-based convolutional neural network (CNN) (ResNet-50) with majority-vote aggregation; (2) a Mixture-of-Experts (MoE) model with learned slice weighting; and (3) a hybrid CNN–Transformer integrating per-slice features across the volume. Two ophthalmologists masked to clinical and genetic data graded the 80 test volumes.

**Main Outcome Measures:**

Volume-level accuracy and weighted F1-score.

**Results:**

On the independent test set (80 volumes: 33 *CHM*, 29 *USH2A*, 18 controls), all models achieved high performance (accuracy ≥0.963). The MoE achieved the highest accuracy on the internal test set (accuracy 0.988; 79/80; weighted F1-score 0.988), followed by CNN + vote (accuracy 0.975; 78/80; weighted F1-score 0.975) and the hybrid CNN–Transformer (accuracy 0.963; 77/80; weighted F1-score 0.962). Ophthalmologists achieved lower accuracy (0.875 and 0.863). External validation on an independent Rennes University Hospital data set (n = 28 volumes; CHM = 6, controls = 10, *USH2A* = 12), processed identically without retraining, yielded accuracy 28/28 for CNN + vote, whereas the MoE and hybrid CNN–Transformer each achieved 26/28.

**Conclusions:**

Deep learning applied to macular OCT volumes accurately distinguished CHM, USH2A-associated rod–cone dystrophy, and healthy controls, and achieved higher observed accuracy than masked human readers in this OCT-only setting. Both learned slice weighting and simple aggregation achieved strong performance, suggesting that increased architectural complexity may not be necessary for this 3-class classification task. Further validation in larger, more diverse, and clinically representative cohorts is required to determine the potential role of such models as complementary diagnostic support.

**Financial Disclosure(s):**

Proprietary or commercial disclosure may be found in the Footnotes and Disclosures at the end of this article.

Rod–cone dystrophy (historically known as retinitis pigmentosa) is a genetically diverse group of inherited retinal degenerations. Typically, rod dysfunction predominates early on, causing nyctalopia and progressive constriction of the peripheral visual field.^1^ Subsequently, cone dysfunction and loss contribute to impaired central vision and color perception. Cone involvement may reflect the direct effect of the pathogenic variant causing the condition, as well as secondary mechanisms related to rod degeneration, including the loss of rod-derived trophic support.^2^

Usher syndrome is the most common form of syndromic rod–cone dystrophy. Several subtypes are recognized. Type 2 Usher syndrome is characterized by congenital sensorineural hearing loss of variable severity, which is usually stable or slowly progressive.^3^ Retinal degeneration becomes symptomatic in adolescence or early adulthood. The majority of cases are caused by biallelic pathogenic variants in the *USH2A* gene, while *ADGRV1* and *WHRN* account for a smaller proportion of cases.

Choroideremia (CHM) is a rare, inherited, X-linked retinal degeneration caused by pathogenic variants in the *CHM* gene.^4^ It primarily affects the retinal pigment epithelium and choroid, resulting in secondary photoreceptor loss. Clinically, it is a choroidopathy characterized by distinctive changes to the fundus (early “salt-and-pepper” mottling progressing to peripheral chorioretinal atrophy), which is typically accompanied by prolonged preservation of the central island, relatively preserved retinal vessel caliber, and prominent choroidal involvement on OCT. Although it represents a distinct clinical entity, it is often grouped with rod–cone dystrophy due to its inherited retinal degeneration profile.

The advent of molecular therapies has offered new hope for patients with inherited retinal diseases. Notably, the first approved retinal gene augmentation therapy for biallelic pathogenic variants in *RPE65* has demonstrated significant clinical benefits.^5^^,^^6^ However, gene augmentation for *USH2A*-related disease is complicated by the large size of the gene, and alternative strategies are being investigated, including dual-vector approaches and antisense oligonucleotide therapy (e.g., ultevursen/QR-421a).^7^ In CHM, adeno-associated virus mediated *CHM* gene augmentation has been evaluated in clinical trials with mixed results, resulting in an evolving therapeutic landscape.^8^

Due to their rarity, inherited retinal dystrophies (IRDs) remain challenging to diagnose. Nearly one-third of patients with IRDs initially receive an incorrect diagnosis, the average time to correct diagnosis spans several years, and ≥3 clinical visits are typically required to reach the final diagnosis.^9^^,^^10^ Accurate recognition of the dystrophy and identification of the causative gene are important for potential inclusion in gene therapy or other clinical trials.

Rapid progress in artificial intelligence (AI) and machine learning for medical image analysis is facilitating the development of diagnostic support tools. In IRDs, such tools are not intended to replace clinical assessment, multimodal imaging, or molecular testing but may eventually complement these investigations by identifying imaging patterns suggestive of inherited retinal disease or by helping prioritize differential diagnoses.

Several deep learning models have demonstrated strong performance for common retinal diseases such as age-related macular degeneration, glaucoma, and diabetic retinopathy using OCT.^11^, ^12^, ^13^ Inherited retinal dystrophies pose distinct challenges for AI, including their rarity, genetic heterogeneity, and overlapping phenotypes, which have limited the size and breadth of available training data.^14^, ^15^, ^16^, ^17^ Recently, multimodal frameworks such as Eye2Gene have demonstrated the potential of multimodal retinal imaging, including OCT, to support gene-level prioritization across numerous IRD genotypes.^18^ However, focused approaches evaluating the information contained in macular OCT volumes alone remain comparatively underexplored, particularly for specific classification tasks such as distinguishing CHM from USH2A-associated rod–cone dystrophy. Recent deep learning approaches in OCT increasingly leverage the full macular volume rather than a single foveal slice, because diagnostically relevant features may be present only on a subset of B-scans. Volume-level integration can be achieved through several paradigms: (1) per-slice 2-dimensional convolutional neural network (CNN) classification with majority voting, (2) adaptive slice weighting such as Mixture-of-Experts (MoE) models,^19^^,^^20^ or (3) hybrid CNN–Transformer architectures that learn interslice relationships.^21^^,^^22^ Although end-to-end 3-dimensional CNNs and cube-level Vision Transformers have been investigated, they generally involve greater data requirements and computational cost; accordingly, our work focused on the 3 aforementioned 2-dimensional–based volumetric strategies.

The primary aim of this study was to evaluate whether deep learning models could accurately differentiate CHM, *USH2A*-associated rod–cone dystrophy, and healthy controls using macular OCT volumes. As a secondary objective, we compared the performance of 3 different architectural strategies—a slice-based CNN with volume-level aggregation, a MoE model, and a hybrid CNN–Transformer—to determine which approach was most effective in this task. Finally, to contextualize model performance in this controlled OCT-only setting, we compared the models with the diagnostic performance of experienced ophthalmologists who were masked to clinical, multimodal imaging, and genetic information.

## Methods

This retrospective, monocentric study was conducted at Montpellier University Hospital. All macular OCT scans were acquired using a Heidelberg Spectralis system between 2012 and 2021. Raw E2E files were extracted using the OCT-converter Python library.^23^ All data were anonymized, and written informed consent was obtained. The study adhered to the Declaration of Helsinki and was approved by the Ethics Committee of the French Society of Ophthalmology (IRB 00008855).

Patients with genetically confirmed CHM (pathogenic variants in *CHM*) or Usher syndrome type 2A (pathogenic variants in *USH2A*) were included, along with healthy control participants without ocular disease. The age at OCT acquisition and sex were recorded. Disease severity was not systematically recorded and was therefore not used for patient stratification.

All available OCT volumes comprising 19 or 25 B-scans were included, irrespective of whether they corresponded to an initial, intermediate, or later follow-up visit. No preferential selection was made according to visit timing or presumed disease stage. When several volumes were available for the same patient, all volumes from that patient were assigned to the same data set subset.

The acquisition protocol comprising 19 B-scans was the most frequently represented protocol in the retrospective cohort, while the second main protocol comprised 25 B-scans. To retain the largest possible number of eligible examinations while standardizing the input dimensions across the 3 evaluated architectures, all volumes were standardized to 19 B-scans. For acquisitions comprising 25 B-scans, the central 19 scans were retained by removing the 3 most superior and 3 most inferior scans. Native interscan spacing was not resampled or otherwise harmonized across acquisition protocols. Each B-scan was converted to grayscale, replicated to 3 channels, resized to a square matrix (224×224 pixels, scaled to [0–1]), and normalized using ImageNet’s mean and standard deviation (SD). Training augmentation included random horizontal flips and small rotations (±5°). The OCT B-scans were the only images provided to the models, and the associated infrared reflectance and fundus autofluorescence images were not used.

To avoid data leakage, the data set was split at the patient level into training (76%), validation (12%), and test (12%) subsets.

We evaluated 3 strategies for volume-level classification (CHM/USH2A/healthy): (1) a slice-based ImageNet-pretrained ResNet-50^24^ with volume prediction by majority vote across 19 slices; (2) a MoE model using the same ResNet-50 per slice, combined with a router learning slice weights and producing a weighted sum of slice-level predictions;^19^ and (3) a hybrid CNN–Transformer model,^22^ where ResNet-50 features were projected (2048→512) and input as tokens to a 1-layer, 8-head Transformer encoder, with a learnable classification token for volume-level prediction.

**Figure 1:**
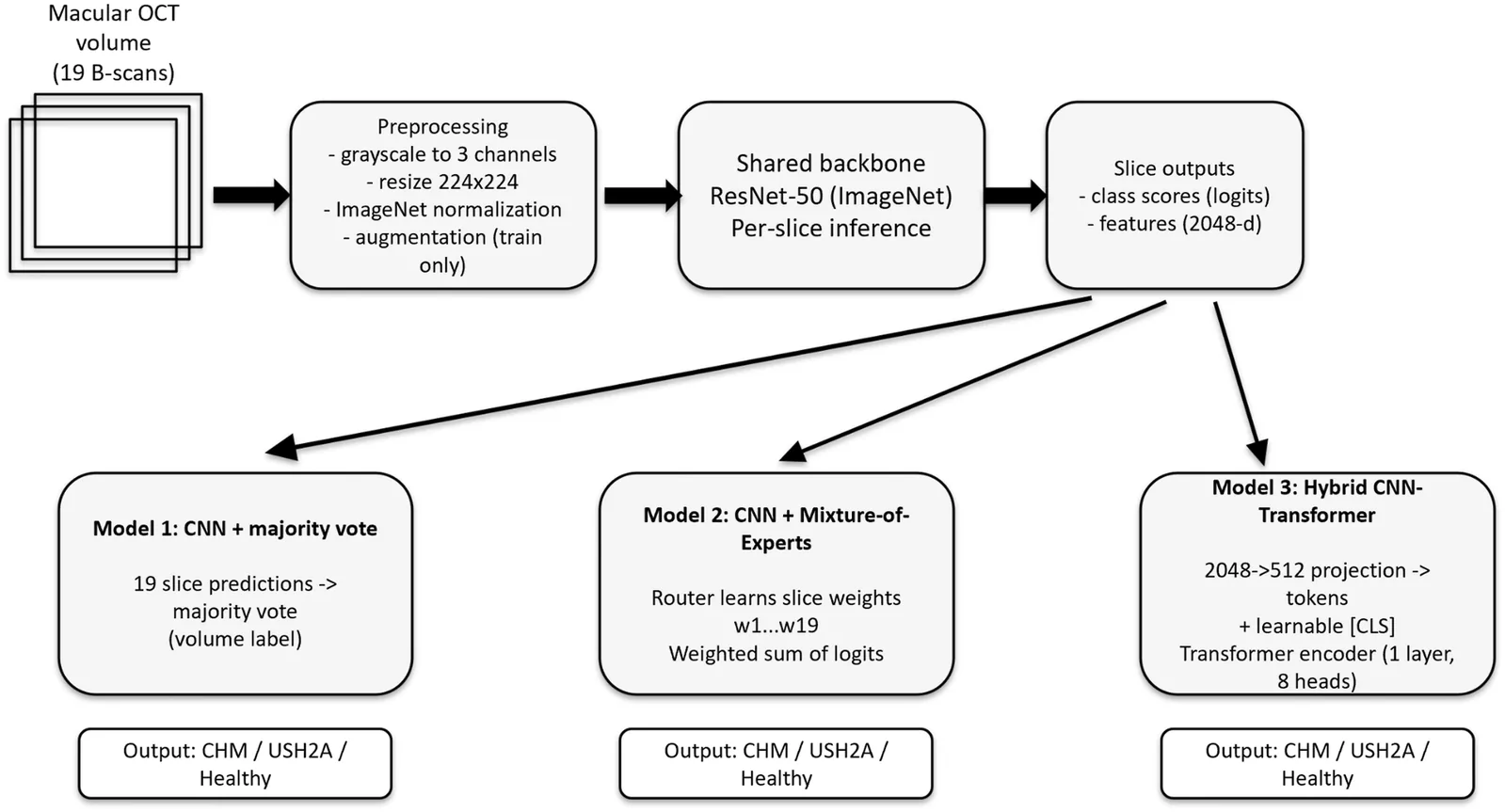
Volumetric OCT classification pipelines evaluated in this study. A macular OCT volume (19 B-scans) is preprocessed (grayscale replicated to 3 channels, resized to 224×224, ImageNet normalization; data augmentation applied during training only) and passed through a shared ResNet-50 backbone (ImageNet-pretrained) to produce per-slice outputs (class logits and 2048-dimensional features). Volume-level prediction is then obtained using one of 3 aggregation strategies: (1) CNN + majority vote across the 19 slice predictions; (2) CNN + Mixture-of-Experts, where a router learns slice weights and computes a weighted sum of slice logits; and (3) hybrid CNN–Transformer, where per-slice features are projected to 512-D tokens and fused by a Transformer encoder with a learnable [CLS] token to yield the final volume label (CHM, USH2A, or healthy). All models share the same per-slice ResNet-50 backbone; only the volume level aggregation differs. CHM = choroideremia; CLS = classification; CNN = convolutional neural network; USH2A = Usher syndrome type 2A.

These 3 architectures represent complementary strategies for volumetric OCT analysis: per-slice classification, adaptive expert weighting, and contextual modeling across slices (Fig 1).

All models were trained in PyTorch using the same protocol (AdamW optimizer; learning rate 1.5 × 10^–4^; weight decay 1 × 10^–4^; 35 epochs; weighted cross-entropy loss; batch size 4; mixed-precision training; ReduceLROnPlateau scheduler). The best checkpoint was selected by validation loss. Deterministic seeds were used across NumPy and PyTorch. Experiments ran on a single NVIDIA RTX 4050 graphics processing unit.

Model performance was assessed at the volume level on the independent test set (n = 80) using accuracy, precision, recall, F1-score, average precision, and confusion matrices. Accuracy was defined as the proportion of volumes that were correctly classified. Precision (positive predictive value) was defined as the proportion of predicted positives that were true positives. Recall (sensitivity) was defined as the proportion of true positives that were correctly identified. The F1-score was defined as the harmonic mean of precision and recall. Precision, recall, and the F1 score were computed per class and reported as support-weighted averages. Average precision was defined as the area under the precision–recall curve, reported as a one-vs-rest macro-averaged metric. Robustness was evaluated with stratified fivefold patient-level cross-validation (reported as mean ± SD). External validation was performed on an independent data set from Rennes University Hospital, processed with the same pipeline and evaluated without retraining when E2E files were available. Two ophthalmologists (N.S. and P.R.), masked to clinical information, genetic results, and all imaging modalities other than the OCT volumes presented for grading, independently graded the 80 test volumes. For patients with bilateral OCT volumes in the internal test set, we also assessed intereye agreement by determining whether both eyes received the same predicted class for each model. Age was compared across groups using 1-way analysis of variance (or Kruskal–Wallis when appropriate) and sex distribution using chi-square testing. We assessed interpretability using perturbation-based occlusion sensitivity. For each OCT volume (19 B-scans), we defined a volume-level target score as the target-class logit (by default, the predicted class) after the model’s native slice aggregation. For the CNN + majority vote model, occlusion sensitivity used the mean slice logits as a continuous volume score (final classification used majority vote). Slice importance was computed with a leave-one-slice-out procedure by replacing each slice with a baseline (mean of the remaining slices; alternatively zero) and measuring the resulting drop in the volume-level target logit (normalized). For local attribution, we applied patch-wise occlusion on the most influential slice using a regular grid (e.g., 12×12), masking each patch with the slice’s per-channel mean and recording the induced change in the volume-level target logit (upsampled for visualization). We chose occlusion sensitivity because it is architecture-agnostic and directly reflects changes in the final aggregated decision.

## Results

### Study Cohort

A total of 535 macular volumes (10 165 B-scans) were included: 210 volumes from 99 patients with CHM; 195 volumes from 96 patients with *USH2A*-related rod–cone dystrophy (USH2A); and 130 volumes from 67 healthy controls (Table 1). As expected for an X-linked disorder, the CHM cohort was predominantly male.

**Table 1:** Demographic and Clinical Characteristics of the Study Cohort

Group	N patients	Age, Mean ± SD (years)	N volumes	Female (n)	Male (n)
CHM	99	34.3 ± 14.8	210	5	94
Healthy	67	37.4 ± 15.7	130	54	13
USH2A	96	39.8 ± 14.7	195	52	44

CHM = choroideremia; SD = standard deviation; USH2A = Usher syndrome type 2A.

Values are expressed as mean ± SD unless otherwise indicated.

### Volume-Level Diagnosis on an Independent Test Set (n = 80)

The independent test set comprised 80 OCT volumes from 39 patients: 33 CHM volumes, 29 USH2A volumes, and 18 healthy-control volumes. After grouping repeated examinations from the same eye, these 80 volumes represented 72 unique eyes. Thirty-three patients contributed bilateral data (66 patient-eyes), whereas 6 contributed data from 1 eye only. Seven patient-eyes had >1 OCT volume available.

**Figure 2:**
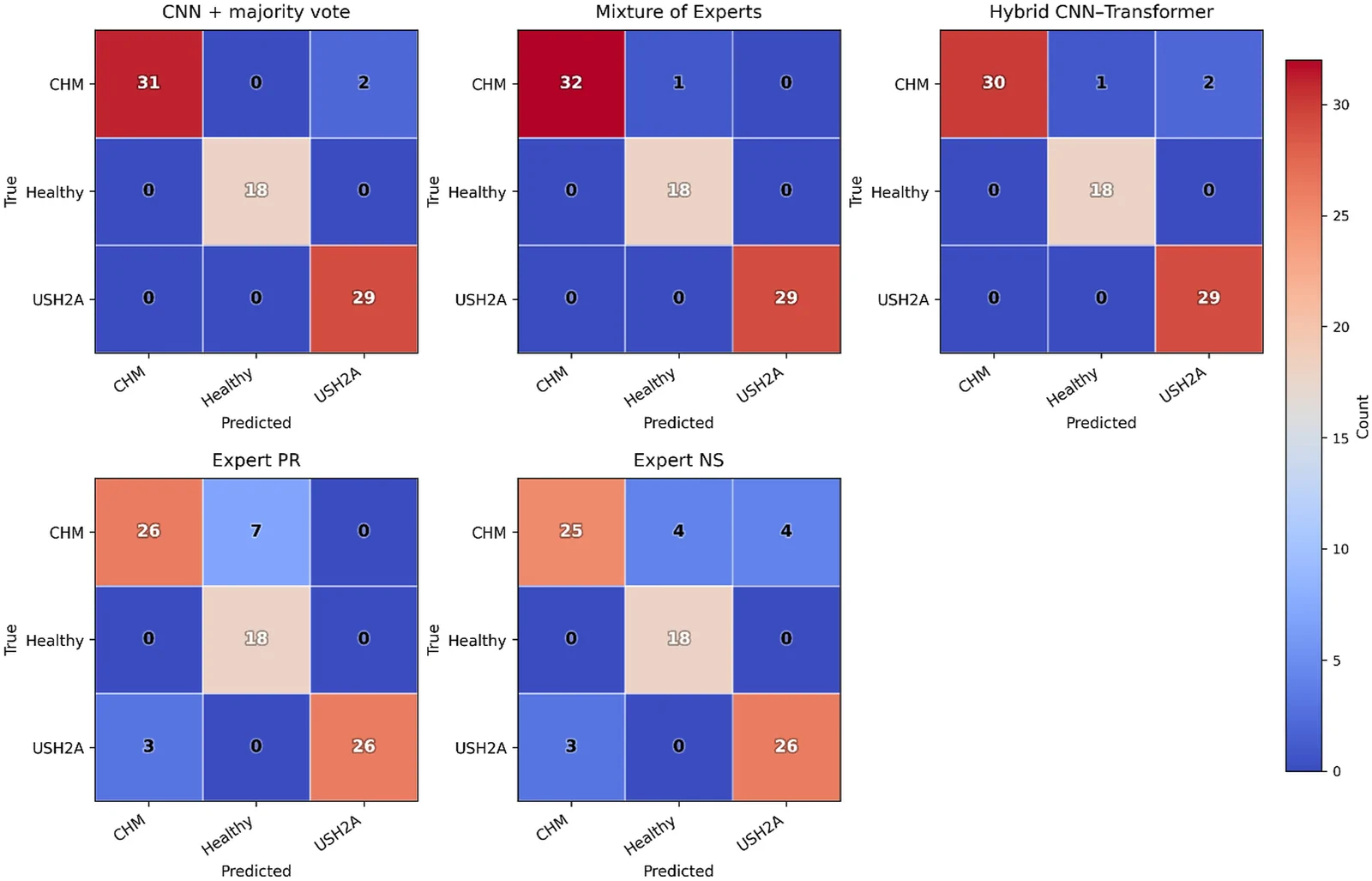
Confusion matrices on the independent test set (n = 80). CHM = choroideremia; CNN = convolutional neural network; USH2A = Usher syndrome type 2A.

The primary outcome was volume-level classification corresponding to 1 prediction per macular OCT volume. All deep learning models achieved high performance at the volume level (accuracy ≥0.963). The MoE model yielded the highest test set accuracy (0.988; 79/80 correct), achieving a weighted F1 score of 0.988. The CNN with majority voting across slices achieved an accuracy of 0.975 (78/80) and a weighted F1-score of 0.975. The hybrid CNN–Transformer model achieved an accuracy of 0.963 (77/80) and a weighted F1-score of 0.962 (Table 2). Figure 2 shows the confusion matrices on the test set.

**Table 2:** Volume-Level Performance on the Independent Test Set (n = 80)

Model/Reader	N	Accuracy	Precision (Weighted)	Recall (Weighted)	F1-Score (Weighted)	Mean Time (s/Volume)
CNN + majority vote	80	0.975	0.977	0.975	0.975	
Mixture of Experts	80	0.988	0.988	0.988	0.988	
Hybrid CNN–Transformer	80	0.963	0.965	0.963	0.962	
Expert PR	80	0.875	0.894	0.875	0.877	3.280
Expert NS	80	0.863	0.867	0.863	0.860	7.570

CNN = convolutional neural network.

### Intereye and Repeated-Volume Agreement

Among the 33 patients with bilateral OCT data, both eyes received the same predicted class in 32/33 patients (97.0%) with the CNN with majority voting, 32/33 patients (97.0%) with the MoE model, and 31/33 patients (93.9%) with the hybrid CNN–Transformer. In all discordant cases, one eye was correctly classified and the fellow eye was misclassified; no patient had both eyes incorrectly classified.

Among the 7 patient eyes with >1 available OCT volume, predictions were identical across repeated volumes in 6/7 patient eyes (85.7%) with the CNN with majority voting, 7/7 (100%) with the MoE model, and 6/7 (85.7%) with the hybrid CNN–Transformer. For each model, no repeatedly imaged eye had >1 misclassified volume.

### Model Interpretability

To characterize which regions of the OCT volumes drove the models’ decisions, we performed a perturbation-based occlusion sensitivity analysis. Attribution maps suggested that predictions for *USH2A*-associated rod–cone dystrophy were predominantly driven by the outer retinal bands, whereas CHM predictions showed greater attribution to the choroid/retinal pigment epithelium complex (Figs S1–S3, available at www.ophthalmologyscience.org).

### Performance of Masked Ophthalmologist Readers in the OCT-Only Setting

Two ophthalmologists (P.R. and N.S.) masked to clinical information, genetic results, and all imaging modalities other than the OCT volumes presented for grading, independently classified the same 80 test volumes. P.R. achieved an accuracy of 0.875 (70/80, with an average reading time of 3.28 seconds per volume), and N.S. achieved an accuracy of 0.863 (69/80, with an average reading time of 7.57 seconds per volume). Both readers correctly identified all healthy controls, with most errors occurring in CHM, which was frequently misclassified as healthy. In this controlled OCT-only setting, all 3 deep learning models achieved higher accuracy than both readers, with an absolute difference of approximately 8–12 percentage points, primarily because of fewer CHM misclassifications (Table 2 and Fig 2).

### Secondary Analysis: Slice-Level CNN Performance

To ensure completeness, the performance of the 2-dimensional CNN on individual B-scans (n = 1520) was also examined. The results showed high accuracy (0.960) and macro F1 (0.961). However, because single-slice interpretation is not the clinical endpoint, the emphasis is on the volume-level results as the primary outcomes (Table S1, available at https://www.ophthalmologyscience.org).

### Robustness: Fivefold Patient-Level Cross-Validation

**Figure 3:**
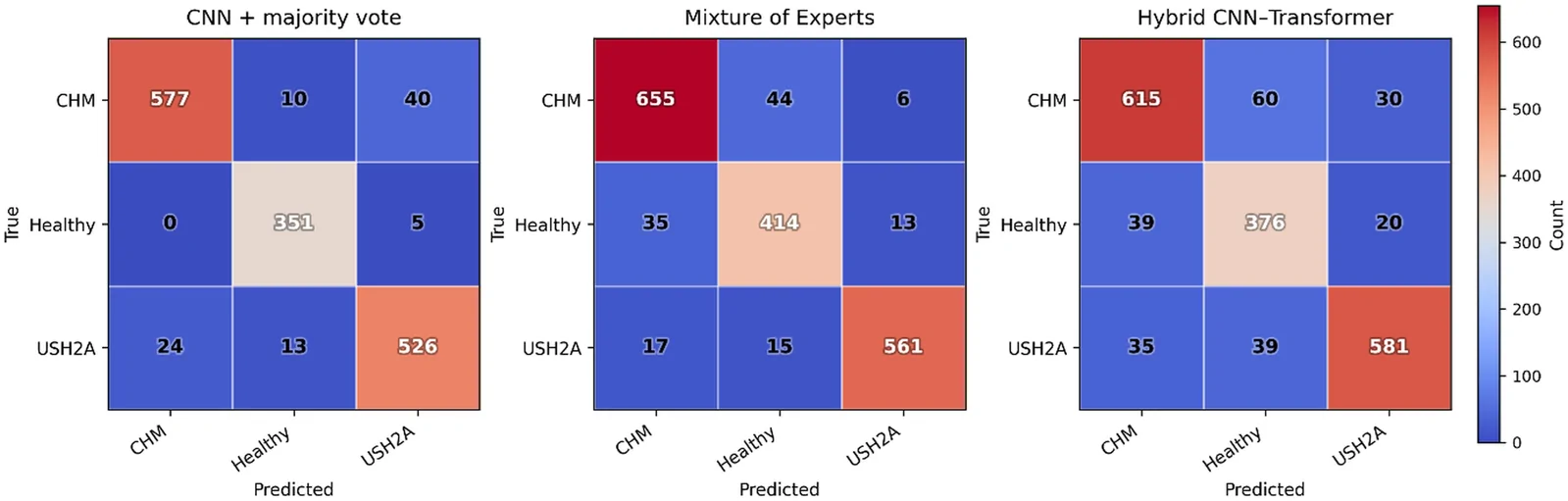
Cumulative confusion matrices across fivefold patient-level cross-validation. CHM = choroideremia; CNN = convolutional neural network; USH2A = Usher syndrome type 2A.

In fivefold patient-level cross-validation, the CNN + vote and MoE showed the most stable performance (SD ˜ 0.03), whereas the hybrid CNN–Transformer exhibited higher variability (SD ˜ 0.065) including one low-performing fold (accuracy 0.822) (Table 3; Table S2, available at https://www.ophthalmologyscience.org). Most errors involved CHM–USH2A confusion, with fewer errors involving healthy controls (Figure 3).

**Table 3:** Patient-Level Cross-Validation Performance (Fivefolds; Mean ± SD)

Model	Accuracy (Mean ± SD)	Precision (Weighted; Mean ± SD)	Recall (Weighted; Mean ± SD)	F1-Score (Weighted; Mean ± SD)	Average Precision (Mean ± SD)
CNN + majority vote	0.9607 ± 0.0260	0.9626 ± 0.0270	0.9607 ± 0.0260	0.9608 ± 0.0260	0.9929 ± 0.0083
Mixture-of-Experts	0.9559 ± 0.0300	0.9574 ± 0.0322	0.9559 ± 0.0300	0.9560 ± 0.0300	0.9894 ± 0.0107
Hybrid CNN–Transformer	0.9366 ± 0.0646	0.9404 ± 0.0700	0.9366 ± 0.0646	0.9373 ± 0.0639	0.9811 ± 0.0290

CNN = convolutional neural network; SD = standard deviation.

### External Validation

External validation was performed on an independent data set from Rennes University Hospital comprising 28 macular OCT volumes (CHM = 6, healthy = 10, and USH2A = 12) using the same preprocessing and inference pipeline without retraining. On this data set, CNN + majority vote achieved 100% accuracy (28/28) with a weighted F1-score of 1.0, whereas MoE and the hybrid CNN–Transformer achieved 92.9% accuracy (26/28) (weighted F1-score 0.929 and 0.927, respectively). Detailed results, including confusion matrices, are provided in Tables S3–S4, available at https://www.ophthalmologyscience.org.

## Discussion

We show that deep learning applied to macular OCT volumes can accurately distinguish CHM, USH2A-associated rod–cone dystrophy, and healthy controls, in this 3-class setting on an independent test set. At the volume level (1 OCT cube = 1 diagnostic decision), all 3 strategies performed highly (accuracy ≥0.963), with MoE achieving the best test performance (0.988; 79/80), followed by CNN + majority vote (0.975; 78/80) and the hybrid CNN–Transformer (0.963; 77/80). Cross-validation further supported robustness, with lower fold-to-fold variability for CNN + vote and MoE than for the hybrid model.

The models' added value over human readers was primarily driven by improved recognition of CHM. On the independent test set, both ophthalmologists correctly classified all healthy controls, whereas most misclassifications involved CHM, which was often incorrectly labeled as normal. This error pattern was substantially reduced by the models. These findings were obtained in a controlled OCT-only setting and should not be interpreted as demonstrating superiority over clinical assessment, which integrates patient history, fundus examination, multimodal imaging, and genetic information. Rather, these results indicate that automated analysis can extract discriminative information from macular OCT volumes alone that may not be consistently recognized by human readers when the same restricted information is provided. As a retrospective evaluation of model performance, this study does not measure direct changes in diagnostic timelines or clinical triage workflows. The practical utility of these models as complementary decision support must therefore be established through prospective investigation under routine clinical practice.^9^^,^^10^

Architecturally, increasing complexity did not consistently improve generalization. The hybrid CNN–Transformer showed greater variability across folds, suggesting that for this 3-class task, strong per-slice features with simple (vote) or learned (MoE) aggregation may be sufficient.^20^^,^^25^^,^^26^ More broadly, IRD-focused AI has historically been limited by rarity and phenotypic overlap,^27^ although recent multimodal, large-scale frameworks for gene prioritization from retinal imaging (e.g., Eye2Gene^18^) illustrate rapid progress in the field. Our study addresses a narrower and complementary question by isolating the discriminative information contained in macular OCT volumes for a specific classification task. The present model does not identify pathogenic variants and cannot be extrapolated to prediction of the causal gene across the full spectrum of inherited retinal diseases. Molecular diagnosis, whether based on targeted gene panels, exome sequencing, genome sequencing, or complementary analyses, remains essential for confirmation and treatment decisions. Imaging-based AI could potentially complement this process by recognizing an evocative phenotype or prioritizing a limited differential diagnosis, but not by replacing molecular testing.

This study has limitations. It is retrospective and largely based on a single acquisition ecosystem (Heidelberg Spectralis), warranting broader multicenter, multidevice validation. Although an independent external data set was included, its limited size warrants cautious interpretation. Disease severity was not systematically recorded or graded. All eligible OCT volumes were included irrespective of whether they were acquired during an initial, intermediate, or later visit, but model performance was not stratified according to disease stage. Consequently, this study cannot establish performance in very early disease, mild disease, or cases with limited macular involvement. Furthermore, because the cohort was derived from a tertiary inherited retinal disease center, its severity distribution may differ from that encountered in general ophthalmology practice. The classification task was restricted to CHM, USH2A-associated rod–cone dystrophy, and healthy controls. It therefore does not represent an inherited retinal disease screening population or the broader differential diagnosis encountered in clinical practice. A clinically useful system would need to include multiple inherited retinal diseases at mild, moderate, and advanced stages, as well as acquired retinal diseases and other relevant mimics in an unselected population. Input volumes were standardized to 19 B-scans, the predominant acquisition protocol in this retrospective cohort; because different scan densities and interscan spacings were not compared, the optimal acquisition protocol for this task remains undetermined. Because CHM is X-linked, our cohorts showed marked sex imbalance; although sex was not provided as an explicit input, residual confounding cannot be fully excluded, particularly because macular OCT can carry a detectable (imperfect) sex-related signal.^28^ Interpretability was assessed using perturbation-based occlusion sensitivity analyses. Although these architecture-agnostic maps yielded anatomically plausible attributions in outer retinal and choroidal regions, they should be regarded as plausibility checks rather than evidence of causal reasoning. They therefore cannot definitively exclude residual demographic confounding or other shortcut learning. Future work should include larger multicenter and multidevice cohorts, additional genotypes and clinically relevant differential diagnoses, severity-stratified early and mild disease, and integration of clinical and multimodal imaging data. Model calibration, uncertainty estimation, and prospective reader-assist studies in representative clinical populations will also be required before any impact on diagnostic pathways can be established.^29^^,^^30^

In summary, when applied to macular OCT volumes, deep learning can accurately distinguish between CHM, *USH2A*-related rod–cone dystrophy, and healthy controls. This was demonstrated through strong performance on an independent test set and robust cross-validation. Compared to human readers, the main added value was improved recognition of CHM in an OCT-only setting. Among the evaluated strategies, simpler volumetric aggregation (majority vote or learned slice weighting) matched or exceeded the performance of the more complex hybrid CNN–Transformer model, suggesting that increased architectural complexity is not required for this 3-class classification task. Further multicenter and multidevice validation, expansion to additional differential diagnoses and genotypes, and prospective reader-assist studies are needed to confirm generalizability and clinical impact.

## Declaration of Generative AI and AI-Assisted Technologies in the Writing Process

During the preparation of this work, the authors used ChatGPT (OpenAI) to improve the clarity and readability of the English language. After using this tool, the authors reviewed and edited the content as needed and take full responsibility for the content of the published article.
