# Supplementary material for: Deep Learning for Diagnosis of Choroideremia and USH2A-Associated Rod–Cone Dystrophy Using Macular OCT Volumes
Source: Ophthalmol Sci. 2026 Aug 20;6(10):101378. doi: 10.1016/j.xops.2026.101378 (PMC13595082; doi:10.1016/j.xops.2026.101378)

Supplementary Figure S1. Occlusion sensitivity maps — CNN (slice aggregation)

Occlusion sensitivity examples for the CNN with slice aggregation (three correctly classified test volumes per class): normalized slice importance (19 B-scans) and patch-wise occlusion map on the most influential slice.

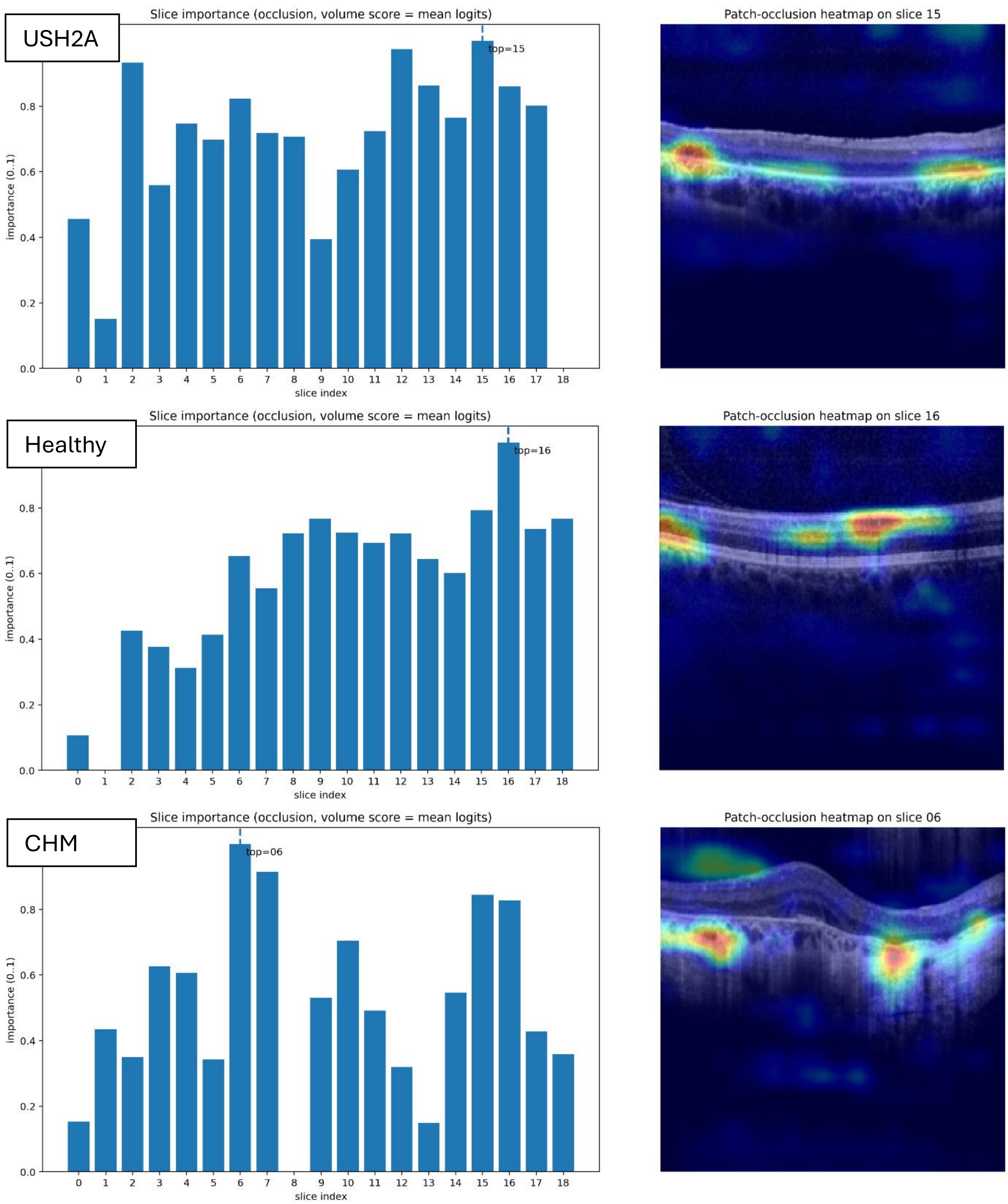

Supplement: Supplementary file 1 [file mmc1.pdf]
