# Supplementary material for: Deep Learning for Diagnosis of Choroideremia and USH2A-Associated Rod–Cone Dystrophy Using Macular OCT Volumes
Source: Ophthalmol Sci. 2026 Aug 20;6(10):101378. doi: 10.1016/j.xops.2026.101378 (PMC13595082; doi:10.1016/j.xops.2026.101378)
Supplement: Supplementary file 4 [file mmc4.pdf]

Table S1. Slice-level CNN performance on the independent test set (n=1,520 B-scans).

Metric	Value
Accuracy	0.960
MacroF1	0.961
Weighted F1	0.961

Slice-level evaluation is provided for completeness; the primary endpoint of the study is volume-level classification.
