# Supplementary material for: Deep Learning for Diagnosis of Choroideremia and USH2A-Associated Rod–Cone Dystrophy Using Macular OCT Volumes
Source: Ophthalmol Sci. 2026 Aug 20;6(10):101378. doi: 10.1016/j.xops.2026.101378 (PMC13595082; doi:10.1016/j.xops.2026.101378)
Supplement: Supplementary file 5 [file mmc5.pdf]

Table S2. Fold-by-fold cross-validation metrics.

CNN + majority vote

Fold	Validation volumes (n)	Accuracy	Precision (weighted)	Recall (weighted)	F1-score (weighted)	Average precision
1	97	0.949	0.953	0.949	0.949	0.998
2	101	0.951	0.951	0.951	0.949	0.985
3	104	0.933	0.938	0.933	0.933	0.982
4	142	0.972	0.972	0.972	0.972	0.999
5	86	1.000	1.000	1.000	1.000	1.000

Mixture of Experts

Fold	Validation volumes (n)	Accuracy	Precision (weighted)	Recall (weighted)	F1-score (weighted)	Average precision
1	117	0.974	0.976	0.974	0.974	1.000
2	83	0.904	0.915	0.904	0.903	0.972
3	146	0.966	0.966	0.966	0.966	0.987
4	99	0.960	0.961	0.960	0.960	0.995
5	85	0.977	0.978	0.977	0.976	0.993

Hybrid CNN – Transformer

Fold	Validation volumes (n)	Accuracy	Precision (weighted)	Recall (weighted)	F1-score (weighted)	Average precision
1	79	0.975	0.975	0.975	0.975	0.998
2	88	0.966	0.975	0.966	0.968	0.992
3	107	0.822	0.835	0.822	0.823	0.929
4	138	0.971	0.971	0.971	0.971	0.992
5	118	0.949	0.958	0.949	0.950	0.994
