# Supplementary material for: Deep Learning for Diagnosis of Choroideremia and USH2A-Associated Rod–Cone Dystrophy Using Macular OCT Volumes
Source: Ophthalmol Sci. 2026 Aug 20;6(10):101378. doi: 10.1016/j.xops.2026.101378 (PMC13595082; doi:10.1016/j.xops.2026.101378)
Supplement: Supplementary file 6 [file mmc6.pdf]

Table S3. Volume-level performance on the independent external validation dataset (n=28 volumes). External dataset composition: CHM=6, Healthy=10, USH2A=12. Metrics are computed at volume level. AP = average precision (one-vs-rest).

**Model**	**N volumes**	**Accuracy**	**Precision (weighted)**	**Recall (weighted)**	**F1 (weighted)**	**AP (macro, OvR)**
CNN + majority vote	28	1.0000	1.0000	1.0000	1.0000	1.0000
Mixture of Experts	28	0.9286	0.9286	0.9286	0.9286	0.9899
Hybrid CNN-Transformer	28	0.9286	0.9388	0.9286	0.9274	0.9801
