# Supplementary material for: Deep Learning for Diagnosis of Choroideremia and USH2A-Associated Rod–Cone Dystrophy Using Macular OCT Volumes
Source: Ophthalmol Sci. 2026 Aug 20;6(10):101378. doi: 10.1016/j.xops.2026.101378 (PMC13595082; doi:10.1016/j.xops.2026.101378)
Supplement: Supplementary file 7 [file mmc7.pdf]

Table S4. Confusion matrices on the external validation dataset (rows = true class, columns = predicted class).

A) CNN + majority vote

True \ Pred	CHM	Healthy	USH2A
CHM	6	0	0
Healthy	0	10	0
USH2A	0	0	12

B) Mixture of Experts

True \ Pred	CHM	Healthy	USH2A
CHM	5	0	1
Healthy	0	10	0
USH2A	1	0	11

C) Hybrid CNN-Transformer

True \ Pred	CHM	Healthy	USH2A
CHM	6	0	0
Healthy	0	8	2
USH2A	0	0	12
